# Anaesthesia in Dentistry

**Published:** 1914-08-15

**Authors:** D. Fyfe

**Affiliations:** Glasgow


### ANAESTHESIA IN DENTISTRY.

A REVIEW OF FISCHER'S BOOK, BY D. FYFE, L.D.S., GLASGOW.

One of the most important branches of modern dentistry is that of anaesthesia. Professor Fischer, with that wonderfully patient, scientific outlook and mind so characteristic of German scientists, has brought local anaesthesia in its use for dental purposes to a high degree of perfection. The writer of this review procured on its first issue a copy of the excellent English translation by Dr. Rathmuller of Professor Fischer's second edition of his book on local anaesthesia. He was so struck with the importance of this class of work that he took the first available opportunity (which only occurred last autumn) of visiting and having personal study with Professor Fischer in the University Clinic at Marburg. Several important advances have been made since the English edition was published.

The anaesthetic used is still novocain with a small percentage of synthetic suprarenin. The novocain-suprarenin is used in the form of tabloids. These tabloids must be absolutely white in colour; any darkening or other discoloration must be discarded as dangerous. Many of the adverse reports on novocain are the result of using solutions which have decomposed and become toxic (whether this toxic change is in the the suprarenin or the novocain is not quite clear). The solution used in which these tabloids are dissolved is Ringer's solution, the formula for which is:—

*Ringer's Solution.*

| | |
|---|---|
| Sodium chloride | 0·05 grm. |
| Potassium chloride | 0·002 " |
| Calcium chloride | 0·004 " |
| Distilled water | ad. 10 c.c. |

Thus we see that thymol is no longer used as an ingredient. The solution must be carefully prepared with distilled water, and must be absolutely sterile. The glass of the bottle in which the solution is kept must be alkali-free, otherwise the fluid becomes alkaline. The necessity for the solution being neutral in reaction is seen when one realizes that novocain is slightly acid. If alkaline, a pinkish discoloration results on the addition of the novocain. This must not be injected.

The research work of such men as Fischer has emphasized the enormous importance of absolute asepsis in local anaesthesia. In preparing Ringer's solution the writer has a reliable pharmacist prepare a few ounces guaranteed absolutely sterile. This is kept in a well-stoppered alkali-free glass bottle—the necessary quantity poured into a small thoroughly washed porcelain dish (with a handle). This is thoroughly boiled, then a novocain tabloid added and reboiled. It is wise to remove the stopper from the bottle over a Bunsen flame, thus sterilizing that part over which the solution must flow. Professor Fischer has tabloids prepared for him containing the ingredients of Ringer. He prepares about one ounce of the solution which he keeps in a small bottle. This bottle is kept in a small electric sterilizer, and always brought to the boiling point before the necessary quantity of the fluid is poured into the small mixing dish. He again boils after the novocain tabloid has been added. It is unwise to keep the Ringer solution too long as it tends to decompose. Even the Ringer tabloids are better not used after a few months—completely fresh ones should be made. The novocain solution may be used in the strength of 1 per cent., 1½ per cent., or 2 per cent., depending on the depth of anaesthesia required, also on the physical condition of the patient, a young child or a feeble adult having the lower percentages injected. The treatment of the hypodermic syringe is most important. The needles are made of iridio-platinum and can thus be passed into the flame and rendered absolutely sterile. Fischer has a glass jar (specially made for the purpose) with a tightly fitting glass lid. A metal stand capable of holding three syringes, needles downwards, is fitted into the jar, which is half filled with a solution composed of three parts absolute alcohol to one part pure glycerine (the glycerine absorbs any water that may get into the alcohol). The syringes are kept in this solution. Before being used the syringe must have some boiled water drawn into it to get rid of any sterilizing fluid in the barrel. The iridio-platinum needle is passed into a flame and made red-hot, then placed in the anaesthetic solution and the syringe filled. These iridio-platinum needles are not removed from their syringes until they break or are otherwise rendered useless, when a new one is fitted. The hypodermics used by Fischer are his own design and are excellent, all parts being of glass and metal. The plunger and barrel are so carefully fitted that a high degree of pressure is obtainable, this being necessary when injecting solutions under the periosteum. Sterilization of the gums before injecting is most important. This he still does by the use of tincture of iodine. A pledget of cotton wool on an instrument is saturated with tincture of iodine, and the parts to be sterilized rubbed—not simply swabbed—with the solution. The rubbing is most important as it mechanically cleanses the gums; thus mechanical cleansing plus fixation and destruction of micro-organisms is obtained.

Another point emphasized by Professor Fischer is the use of the neck bandage—so-called stasis bandage. This has been advocated in the English edition of his book. Time and experience have proved the importance of this procedure more particularly with injections used to block the nerve trunks (conduction anaesthesia), such as mandibular injection. In these cases a comparatively large quantity of the solution is thrown into loose tissue, and thus more quickly absorbed. The writer of this review has had a feeling that many of the annoying symptoms observed—when all aseptic precautions have been carefully carried out—such as faintness, quick pulse and semi-hysterical conditions, are due to the suprarenin, not to the novocain. A possible psychic influence must not be overlooked. A London doctor, reporting in the *British Medical Journal* of May 2, 1914, says he gave himself an injection of 3 minims 1 in 1,000 adrenaline chloride solution, which was followed by a feeling of extreme illness. He had quick pulse, shaking, etc. Fischer insists that the neck bandage is a valuable means of lessening many of these troublesome symptoms. It acts by pressure on the superficial veins of the neck, thus lessening the tendency to anaemia of the brain. The technique in conductive and local anaesthesia is unchanged from that found in the English translation. Fischer is a firm believer in conductive mandibular anaesthesia. He has found local anaesthesia of the mandible, more particularly in the premolar and molar regions, most unsatisfactory and almost exclusively uses the conductive method by injecting in the region of the inferior dental foramen. He very often injects into the region of both foramina, thus having complete anaesthesia of entire lower jaw (and part of tongue). The present writer can testify to the efficiency of the anaesthesia. He had a single injection made (*i. e.*, on one side only); within five minutes the entire half of the jaw was absolutely anaesthetized, the edge of the tongue, lip and external aspect of lower jaw being also involved in the anaesthesia. In about an hour sensation was re-established. The injection was quite painless and was followed by no after pain or disagreeable results beyond some anxiety entirely psychic in its origin.

I think it is wise to avoid unnecessary conductive anaesthesia in very highly strung and imaginative people unless the operator be able to stand by and control the workings of the patient's mind. As a local injection in the mandible, novocain is a comparative failure. There is thus still ample field for research in local anaesthetics. It is asserted that novocain is as powerful in its anaesthetic properties as cocain while having only one-sixth of its toxicity. The writer quite believes in the comparative non-toxicity of novocain and thus considers it the only anaesthetic suitable in conductive anaesthesia (combined with the stasis bandage). Any preparation of cocain is positively dangerous when used for conductive work—this due to the amount of the anaesthetic and the laxness of the tissue into which the drug is injected, allowing of quick absorption into the general system. The statement as to its equality with cocain or alypin as an anaesthetic is not shown to be correct in clinical investigation. For satisfactory local anaesthesia of the mandible some other anaesthetic than novocain is necessary. The really ideal local anaesthetic is still to be found. In the maxilla, on the other hand, the results of local anaesthesia with novocain are excellent. The injection must be made under the periosteum and with considerable pressure as explained in the English translation. Fischer uses the same technique as that given in his former books for conductive anaesthesia of the maxilla. He injects with blunt needle into the region of Meckel's ganglion, and may inject both sides of the maxilla, thus giving a most efficient anaesthesia (sufficient to do a serious operation). For simple extractions or pulp work local anaesthesia is so thoroughly satisfactory that the writer has never seen any necessity for the more difficult conductive injection. There are two regions in the maxilla to which special attention may be drawn; although they are really conduction anaesthesia regions, the technique in their injection is as simple as that of the local. One is the region of the large foramina through which branches of the posterior superior dental nerve enter the maxilla. The needle is passed into the tissues at the junction of the gum with the cheek approximately above the second molar. The needle then passes upward and backwards close to the bone, and with the opening of the needle facing the bone behind the zygoma (which should be palpated with a finger). The other region is that of the posterior palatine foramen, which lies closely to the palatine aspect of the last erupted molar. Fischer never injects into the region of the anterior palatine fossa, *i. e.*, immediately behind and between the superior central incisors. The result of such an injection is acute pain with no compensating results. Injection in the region of the infra-orbital foramen for conduction anaesthesia is not very satisfactory. The value of a simple subperiosteal

(local) injection in cases of hyper-sensitive dentine is undoubted, especially in the maxilla. A few minutes (about ten) should elapse from the time of the injection until work on the tooth is attempted. Fischer has nothing to say about so-called intra-alveolar injection. The results of such injections are excellent, and if carried out with the same aseptic precautions as taught by Fischer in his works no one should fear the slightest untoward result. The greatest difficulty in all forms of injection anaesthesia is the preliminary pain of the needle. When such can be overcome we shall have gone far in perfecting anaestheisa. There is still ample field for research in anaesthetic substances. So far we have not found the ideal. To those who have not read Professor Fischer's book on anaesthesia the writer would most heartily recommend the English edition, which is excellently translated. The later developments given in this review will bring the reader up to date in this important subject.—*British Dental Journal—Glasgow.*

